# Phenotypic plasticity in an extended phenotype: the cobweb spider *Campanicola campanulata* alters web architecture in response to variation in prey availability

**DOI:** 10.1093/cz/zoaf073

**Published:** 2025-11-19

**Authors:** Haixin Zhang, He Zhang, Lang Hu, Lelei Wen, Changchun Li

**Affiliations:** Hubei Key Laboratory of Resource Utilization and Quality Control of Characteristic Crops, College of Life Science and Technology, Hubei Engineering University, Xiaogan 432000, Hubei, China; Guo Shoujing Innovation College, Xingtai University, Xingtai 054001, Hebei, China; Hebei Province Sweet Potato Breeding and Application Technology Innovation Center, Xingtai 054001, Hebei, China; Hubei Key Laboratory of Resource Utilization and Quality Control of Characteristic Crops, College of Life Science and Technology, Hubei Engineering University, Xiaogan 432000, Hubei, China; Hubei Key Laboratory of Resource Utilization and Quality Control of Characteristic Crops, College of Life Science and Technology, Hubei Engineering University, Xiaogan 432000, Hubei, China; Hubei Key Laboratory of Resource Utilization and Quality Control of Characteristic Crops, College of Life Science and Technology, Hubei Engineering University, Xiaogan 432000, Hubei, China

**Keywords:** *Campanicola campanulata*, defense, extended phenotypes, foraging, plasticity, tradeoff

## Abstract

Phenotypic plasticity in extended phenotypes has been proposed as a mechanism by which animals modify their phenotypes in response to changing environmental conditions. This phenomenon is exemplified by certain spiders that construct bell-shaped cobwebs using sand grains, soil particles, prey remains, and plant debris. The retreat and gumfooted lines in these cobwebs are believed to serve defensive and foraging functions. This study aimed to test both the phenotypic plasticity and the tradeoff hypotheses by examining whether the common cobweb spider *Campanicola campanulata* alters web architecture and behavioral investments in response to variations in prey availability. When prey availability increases, spiders build webs higher, construct smaller and lighter retreats, and produce cobwebs with fewer gumfooted lines and smaller capture areas. Results indicated that, in the presence of abundant ant-prey, spiders reduced their investment in both foraging and defense. These findings suggest that the web architecture of *C. campanulata* exhibits a high degree of plasticity in response to variation in prey availability, but investment in web architectures does not involve tradeoffs between foraging and defense.

## Teaser

We offer a new model to investigate extended phenotypic plasticity and present a new framework for quantifying energy allocation between foraging and defense in the cobweb-building spider. The results of this study can provide important insights into animal adaptability to environmental pressures and the evolution of their behavioral mechanisms.

## Graphical Abstract

**Graphical Abstract:**
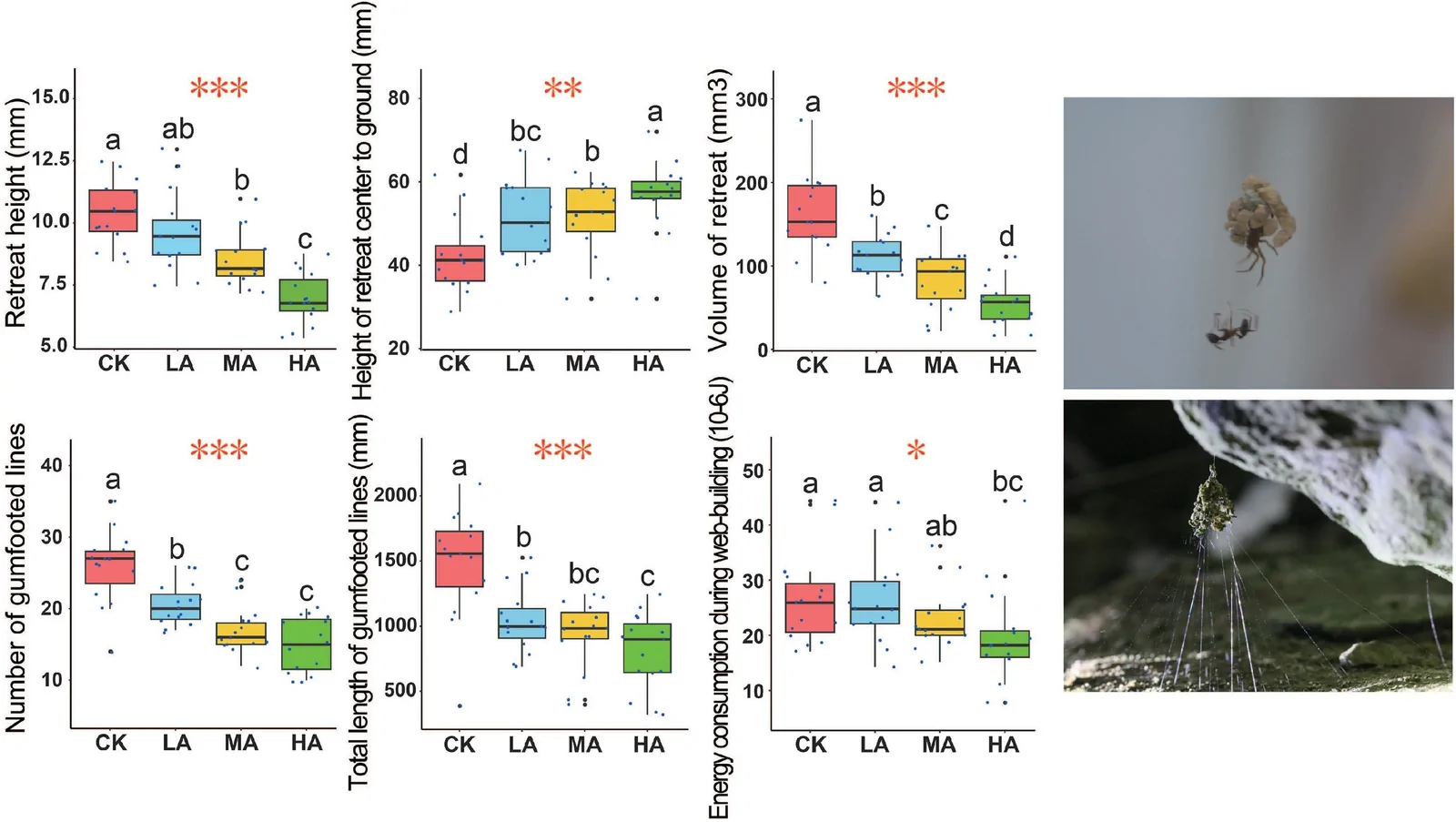

Many animals construct physical structures, such as bird nests, mammal burrows, and spider webs, to serve as foraging devices, mating or breeding platforms, and anti-predator shelters (Blamires 2010; Kelley and Endler 2012; Smith et al. 2015; Wang et al. 2022; Zhang et al. 2023a, 2023b). These structures often reflect behavioral decisions (Blamires et al. 2013) and mediate a variety of fitness-related processes (Pinter-Wollman 2015; Smith et al. 2015). The phenomenon of animals modifying the form or function of their phenotype in response to changing environmental conditions is known as extended phenotypic plasticity (Dawkins and Dennett 1999; Schaedelin and Taborsky 2009; Auld et al. 2010; Scharf et al. 2011). For example, the cobweb spider *Campanicola campanulata* (Araneae: Theridiidae) alters the architecture of its webs in response to differences in detritus weight (Zhang et al. 2023c). Ant-lions (*Myrmeleon crudelis*) increase trap diameter to enhance prey encounter probability when prey is scarce, but deepen pit depth to improve prey retention when prey is abundant (Farji-Brener and Amador-Vargas 2019). Sit-and-wait predators with extended phenotypic plasticity (e.g. ant-lions; web-building spiders) may have selective advantages because they can make rapid behavioral changes in response to prey abundance and maximize fitness in variable environments (Wong and Candolin 2015; Scharf 2016). Therefore, they are excellent model organisms for studying extended phenotypic plasticity induced by prey availability (Olive 1982; Schneider and Vollrath 1998).

Tradeoff theory posits that animals strategically allocate limited resources among competing survival needs by weighing the costs and benefits of different extended phenotypes, ultimately to maximize their fitness (Berger et al. 2014; Embar et al. 2014; Gallant et al. 2016; Pianka 2015; Zhang and Hui 2014). From this perspective, phenotypic plasticity is viewed as an adaptive response that allows organisms to dynamically adjust their phenotypes in the face of conflicting selection pressures, such as the tradeoff between foraging needs and anti-predator defenses driven by environmental factors like prey availability and predation risk (Lima and Dill 1990; Tollrian and Harvell 1999; Whitman et al. 2009; Ellendula et al. 2021). The tradeoff between capturing prey and avoiding predators is thought to influence optimal foraging investment in order to maximize the net foraging gain (Lima and Dill 1990; Ellendula et al. 2021). Specifically, animals should prioritize strengthening the foraging components of the extended phenotype and increasing foraging investment when prey availability is high (Prokopenko et al. 2023). Conversely, animals should enhance the defensive components of the extended phenotype and increase defense investment when predation risk is high (Nakata 2009; Blamires et al. 2017; Ellendula et al. 2021; Mishra and Rastogi 2023). For example, male *Pomatoschistus minutus* reduce the size of their defensive openings in the presence of predators (Lissåker and Kvarnemo 2006). Similarly, *Latrodectus hesperus* reallocates silk from gumfooted threads, which aid in prey capture, to more supportive threads that enhance the stability of the web when prey availability is high (Blackledge and Zevenbergen 2007). However, increased foraging investment may incur costs, such as decreased resource reserves or heightened predation risk due to factors unrelated to foraging. Animals can maximize adaptation to changing environments by expressing different extended phenotypes and strategically balancing their behavioral investment in different contexts (Wong and Candolin 2015; Scharf 2016).

Cobweb spiders (e.g. black widow spiders) are popular candidates for studying extended phenotypic plasticity. Their webs are believed to have a stronger selective advantage more than 2D webs because these webs integrate both gumfooted lines for foraging and retreats for anti-predator defense (Blackledge et al. 2002; Haberkern et al. 2024). Notably, the construction behavior of 3D webs is influenced by both the spider's internal state and external factors (Haberkern et al. 2020; Sergi et al. 2020; Thompson et al. 2020; Zhang et al. 2023a, 2023b). Theoretically, spiders with more gumfooted lines achieve greater foraging success: when prey contacts glue droplets at the base of a gumfooted line, the line's weak pyriform anchor releases. This causes the taut line to contract, suspending the prey and rendering escape attempts ineffective (Zevenbergen et al. 2008; Opell et al. 2024). Meanwhile, retreat construction is well documented to enhance fitness by reducing predation risk (Manicom et al. 2008). These dual functional demands suggest that fitness tradeoffs may occur—where losses from investing in one trait (e.g. foraging) could be offset by gains in another (e.g. defense). Therefore, studying changes in web architecture and behavioral investment in response to different environmental conditions may provide new insights into the extended phenotypic plasticity and behavioral investment strategies of web-building spiders (Zhang et al. 2023b; c). Most research assessing the plasticity of extended phenotypes in cobweb spiders has focused on black widow spiders, which can respond to environmental cues by altering the number and total length of gumfooted threads, the volume of the retreat, or the behavioral decisions during web-building (Blackledge and Zevenbergen 2007; Salomon 2007; Zevenbergen et al. 2008; DiRienzo and Montiglio 2016; DiRienzo and Aonuma 2018; Sergi et al. 2020; Thompson et al. 2020). However, these studies have faced significant challenges and have yielded inconsistent results. The difficulties primarily stem from the challenge of distinguishing between defense and foraging components of the web, as well as accurately measuring the total length, area, and volume of the gumfooted threads (Blackledge and Zevenbergen 2007; Zevenbergen et al. 2008; Salomon 2011; Sergi et al. 2020). For example, studies have shown that black widow spiders exhibit context-specific plasticity in their web-building behavior, but the exact mechanisms and outcomes vary across different populations and environmental conditions.

Spiders in the family Therididae, particularly those in the genus *Campanicola* and *Parasteatoda* (Henschel and Jocqué 1994; Li et al. 2021; Fig. 1), which construct detritus-based, bell-shaped cobwebs, are ideal models for studying web architecture plasticity and investment tradeoff. This is due to the clear separation of defense and foraging components and the precise quantification of behavioral investment during web-building (Zhang et al. 2023a, 2023b, 2023c). These cobwebs typically consist of three components: anchor silk for anchoring, a bell-shaped retreat for defense, and a capture web for foraging. Previous research on these spiders has primarily focused on taxonomy (Yoshida 2015, 2016; Li et al. 2021). Recent studies have demonstrated that this extended phenotype of these spiders is plastic, with changes in internal state and external factors, such as feeding conditions, developmental stage, and detritus weight, leading to adaptive modifications in web architecture and tradeoffs between different behavioral investments (Zhang et al. 2023a, 2023b, 2023c). Although the phenotypic plasticity and behavioral investment strategies of cobweb spiders are poorly documented, studying their responses to variables like prey availability and predator presence can provide valuable insights into their adaptation to environmental pressures and the evolution of these behavioral mechanisms.

**Figure 1:**
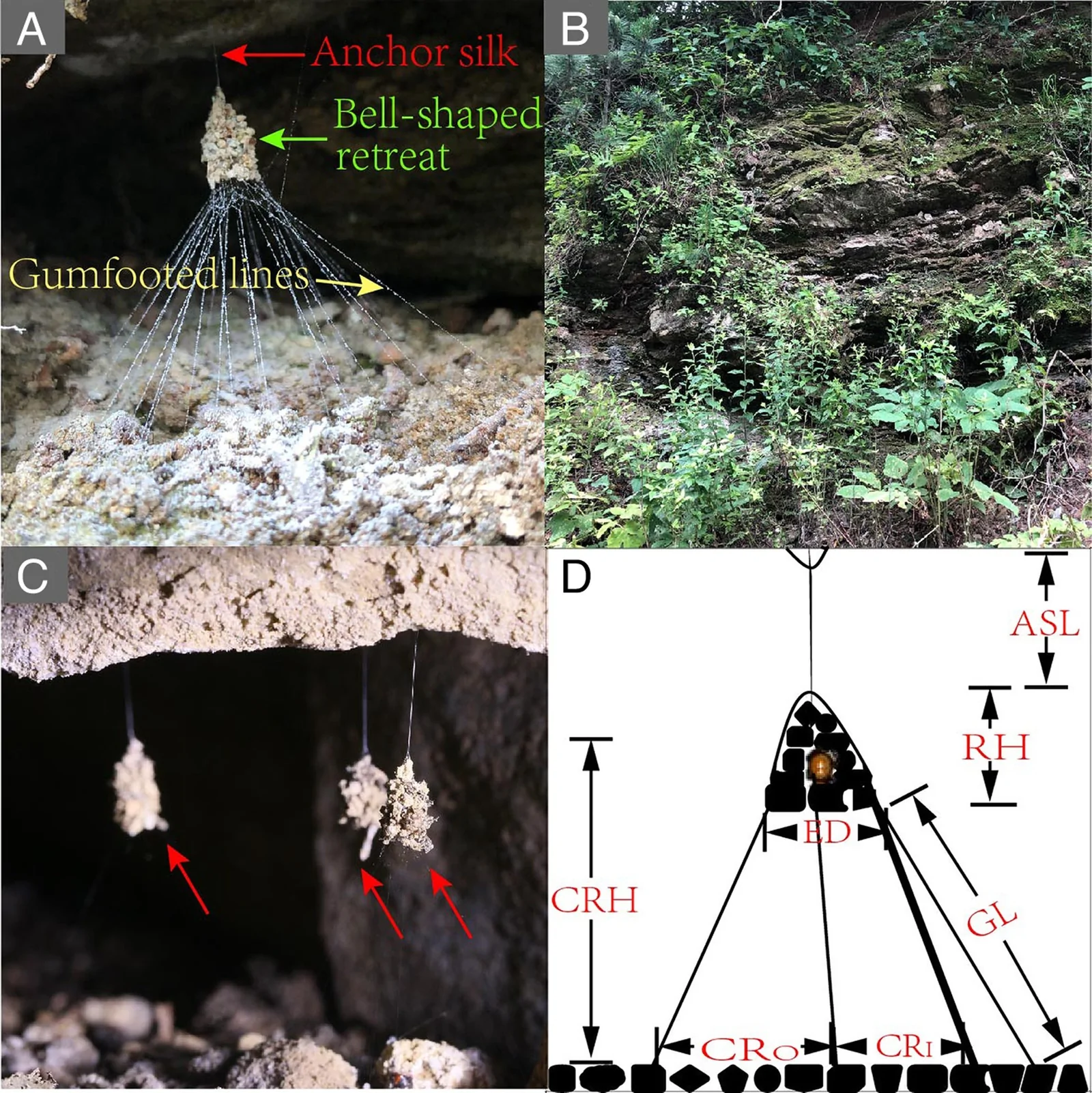
Diagram showing web architecture and behavioral structures of *C. campanulata*, including (A) web components, (B) habitat, (C) group-living pattern, and (D) web measurements used in this study.

Most spiders seem to avoid feeding on ants even though they are among the most common prey, because ants are armed with strong stingers, mandibles, and formic acid, and they can carry out effective collective attacks (Líznarová and Pekár 2013; Pekár and Toft 2015). Therefore, spiders of the genus *Campanicola* that feed on ants are likely to flexibly alter components of their web architectures to trade off different behavioral investment in response to variation in prey availability (Blamires et al. 2014). In this study, we selected the common cobweb spider *Campanicola campanulata*, which constructs detritus-based, bell-shaped retreats, to investigate extended phenotypic plasticity and behavioral investment tradeoffs. We hypothesize that *C. campanulata* adjusts web traits such as gumfooted lines (foraging function) and bell-shaped retreat (defense function) in response to prey availability in order to balance investment in foraging and defense behaviors. This study aims to 1) investigate whether a tradeoff exists between the foraging investment (i.e. total length of gumfooted lines) and the defense investment (i.e. energy consumption during retreat-building) in *C. campanulata*, and 2) test whether *C. campanulata* exhibits plasticity in its web-building behavior. Specifically, we will observe how the spider alters gumfooted line parameters (e.g. number and radiation area) and retreat parameters (e.g. volume and height from the ground) in response to varying levels of ant availability.

## Materials and methods

### Study species

*Campanicola campanulata* (Araneae: Theridiidae) is a cobweb-building spider widely distributed in Hubei, Zhejiang, and Guizhou provinces of China (Chen 1993). These spiders aggregate in cool habitats that are partially sheltered from the rain, primarily feeding on ants (Supplementary Video S1, Supplementary File). We have observed that they often build their webs in locations like concave rocks, earthen walls, or under low-hanging branches. Each individual spider constructs a complete cobweb consisting of anchor silks, dozens of sticky gumfooted lines, and a bell-shaped retreat made of silk, sand, pebbles, debris, leaves, or shells (Supplementary Video S2, Supplementary File).

The anchor silk, composed of strands of silk, connects the bell-shaped retreat to stone walls, tree branches, or other substrates, providing stability and preventing wind damage to the web. The bell-shaped retreat, made of silk-wrapped sand grains, soil particles, prey remains, and plant detritus, is usually suspended and lined with silk tunnels (Henschel and Jocqué 1994). This structure significantly reduces the spider's vulnerability to enemies and provides a safe space for resting, mating, laying eggs, and caring for its young (Henschel and Jocqué 1994). It also offers protection against predators such as araneophagic spiders or birds.

We investigate the hypothesis that *C. campanulata* exhibits phenotypic plasticity and behavioral investment adjustments in response to changes in ant-prey availability by examining the design of its capture web. This web consists of a few to several dozen radial gumfooted lines that extend from the edge of the bell-shaped retreat and are fastened to coarse sand grains or fixed substrates. At the bottom of these lines are glue droplets, which help the spider to trap ant-prey that walk underneath the web and to prevent the intrusion of enemies. Once ants are intercepted by the sticky gumfooted lines, the vibratory signals generated by the ants’ struggles are transmitted to the spider in the retreat. The spider then tears the lines away from the substrate and pulls its prey up toward the retreat, making it difficult for the ant to gain the leverage needed for escape (Argintean et al. 2006).

### Spider collection and maintenance

We collected sub-adult (i.e. 1 molt before adulthood, ∼2.4 mm in body length) *C. campanulata* on March 20 to 25, 2022, at the Guanyin Lake Scenic Area in Hubei Province, China (31˚ 14′ 8.66″N, 114˚ 01′ 21.57″ E), and then cultivated them in the laboratory until they reached adulthood. We chose sub-adult *C. campanulata* for the trial spiders to avoid the influence of their wild mating histories on the experimental results. These spiders were maintained separately in a plastic box (length × width × height: 10 cm × 5 cm × 10 cm) with an inner cavity lined with bamboo strips (providing a support point for the spiders to build their webs) . The front and back of the box were made of removable transparent Perspex glass. All spiders were kept in the laboratory with controlled environmental conditions (temperature: 25 ± 1°C; relative humidity: 80 ± 5%; photoperiod: 14 h:10 h L: D). The bottom of the box was covered with a layer of sand for the spider to collect to build a retreat, and a piece of sponge with absorbed water was placed at the bottom of the box to provide water for the spider. Each spider was fed 2 ants (Monomorium sp., ∼2.2 mm in body length) every 2 days, and the development status (molting) of each spider was checked twice a day (at 09:00 and 21:00, respectively).

### Experimental design and procedure

We selected 60 newly matured female spiders (∼2 days after maturity) that had been well-fed for 3 days and randomly assigned them to four treatments (*N* = 15 per group) with different numbers of ants: blank control (CK)—0 ants, low prey availability (LA)—5 ants, medium prey availability (MA)—10 ants, and high prey availability (HA)—20 ants. Each spider was transferred to a box containing a layer of sand grains at the bottom and a wooden stick attached to the sides and inner wall at the top to form a wooden frame. Sand grains were collected from the wild habitat to provide material for retreat construction, while the wooden frame served as a climbing support for web-building. Before the experiment, we measured the body size (carapace width, body length, and body weight) of each spider. All spiders were provided with *Monomorium* ants and water ad libitum for 2 days prior to the experiment and were given 36 h to build their webs; no spiders were fed during the experiment. Most of the ants remained alive until the end of the experiment. After all the parameters were measured, they were returned to their original wild habitats where they were captured. All trials were recorded using a video camera (HDR-CX 680; SONY).

### Quantification of time parameters for web-building

After being introduced into the experimental setup, the spider undergoes a period of exploration of the new environment, during which it spins silk and fastens it to appropriate sand grains. The spider then climbs to the top of the setup via sticks on both sides and selects a suitable anchor point to begin transporting sand grains. The time from when the spider is introduced into the experimental setup until it begins transporting the first sand grain is defined as the latency of web-building. Due to the energy expenditure required to construct the retreat and gumfooted lines (Zhang et al. 2023a, 2023b, 2023c), the spider takes several rest periods during web-building, but these rest periods did not exceed 2 h each (H. Zhang's pers. obs.). If the spider rests for more than 2 h consecutively, we consider the web-building process to be completed. The period from when the spider begins transporting the first sand grain until web-building is completed is termed the duration of web-building. For each spider, we recorded the latency and duration of web-building by analyzing the videos, as these parameters reflect the spider's decision-making process regarding web-building behavior in the presence of different numbers of ants.

### Measurements of web architecture and behavioral investment

For each web, we measured the parameters of web architecture, including the length of anchor silk (ASL), the height of the retreat (RH), the number of gumfooted lines (GLN), the height of the retreat center from the ground (CRH), the diameter of the retreat entrance (ED), the length of each gumfooted line (GL), and the weight of the retreat (RW) (Fig. 1D). Because the projection of the gumfooted lines on the ground is irregular—with some lines closer to the center and others farther away—we measured the length of the innermost (CR_I_) and outermost (CR_O_) capture radii and used their average as the average radius (CR_A_) to estimate the capture area. The RW was measured after all other web architecture parameters were assessed. We removed the retreat from the web and measured its weight to the nearest 0.01 mg using an electronic balance (FA1004N, HANGPING).

It is well established that the capture area and retreat volume in cobwebs are important indicators of web-building strategy and behavioral investment in capturing prey and avoiding predators. Spiders with larger capture areas typically have higher rates of prey encounter and foraging success (Benjamin and Zschokke 2003; Blackledge and Zevenbergen 2007; Salomon 2007; Zevenbergen et al. 2008; Sergi et al. 2020; Fischer et al. 2023). Conversely, spiders with larger retreats are less likely to be discovered by predators, thereby facing lower predation risk (Manicom et al. 2008). We calculated the capture area of the cobweb and the volume of the retreats with the following formula:


$$\mathrm{Capture}\;\mathrm{area}\;=\pi \times CR^{2}$$

$$\mathrm{Retreat}\;\mathrm{volume}\;=\frac{1}{3}\pi \times {\left(\frac{1}{2}\mathrm{ED}\right)}^{2}\times \mathrm{RH}$$

where


$$\mathrm{CR}=\frac{\left(CR_{I}+CR_{O}\right)}{2}$$

We also quantified foraging investment (i.e. total length of gumfooted lines, GTL) and defense investment (i.e. energy consumption during web-building, EC) with the following formula:


$$\begin{array}{rl} & \mathrm{Total}\;\mathrm{length}\;\mathrm{of}\;\mathrm{gumfooted}\;\mathrm{lines}\;\\ & \;\;\;=\sum GL_{1}+GL_{2}+GL_{3}+\ldots GL_{n} \end{array}$$

Defenseinvestment=RW×CRH×g

where, GL_1_, GL_2_… GL*_n_* are the length of each line in a web of *n* gumfooted lines, *π* is 3.14, and *g* is 9.8.

### Statistical analysis

We assessed the normality and homogeneity of variance of the data using the Shapiro–Wilk and Levene tests, respectively. Because the data were not normally distributed, we used the Kruskal–Wallis test to compare differences in web architecture and behavioral investments among treatments. When significant differences were detected, pairwise comparisons were conducted using the Wilcoxon rank-sum test with Bonferroni correction. All statistical analyses were performed using R version 4.4.2 (R Core Team 2025). All tests were two-tailed, with a significance level set at *P* < 0.05.

## Results

There were no significant differences in body size (carapace width, body length, and body weight) among the 4 treatments (Table 1). In the measurement of web morphology, a total of 60 webs were measured, as all tested spiders successfully built intact webs.

**Table 1:** Mean ± SE of body size for spiders in the study (*N* = 15 in each treatment).

Parameters	CK	LA	MA	HA	*χ^2^*	*df*	*P*
Carapace width (mm)	0.937 ± 0.006	0.938 ± 0.007	0.933 ± 0.007	0.9311 ± 0.006	1.448	3	0.694
Body length (mm)	2.524 ± 0.005	2.519 ± 0.020	2.504 ± 0.021	2.501 ± 0.021	0.142	3	0.986
Body mass (mg)	3.747 ± 0.252	3.960 ± 0.309	3.733 ± 0.319	3.513 ± 0.231	1.248	3	0.742

### Latency and duration of web-building

**Figure 2:**
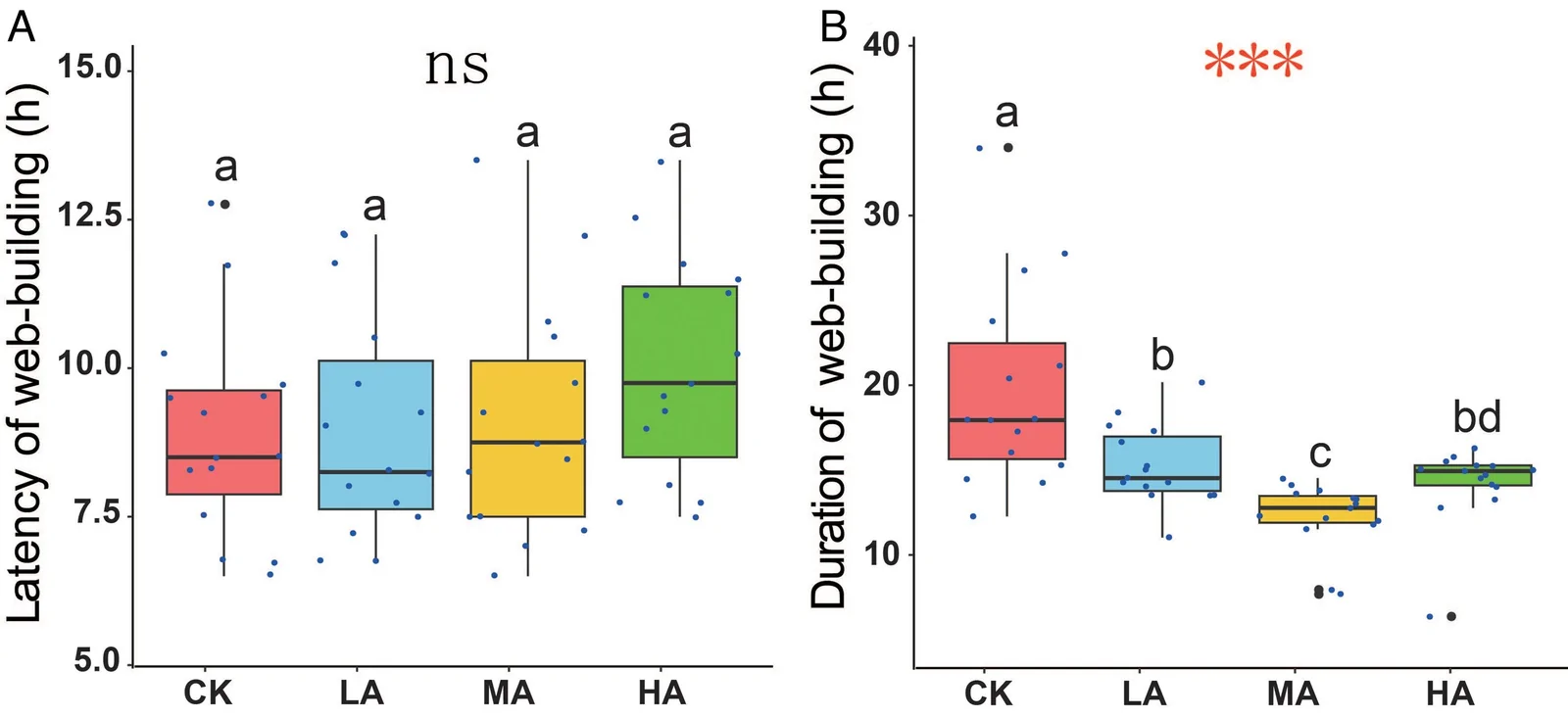
Boxplots of the latency and duration of web-building for *C. campanulata* in different availability of ants: (A) Latency of web-building (LW); (B) duration of web-building (DW). Boxplots show the median (central line), first and third quartiles (box), and different lower-case letters indicate a significant difference between treatments. Ns, no significant difference. ***P < 0.001.

The latency of web-building increased with the availability of ants, but there were no significant differences among the four groups (*χ^2^* = 3.258, *df* = 3, and *P* = 0.354) (Supplementary Table S1; Fig. 2A). In contrast, the duration of web-building decreased with increasing prey availability of ants, and significant differences were observed among the groups (*χ^2^* = 28.01, *df* = 3, and *P* < 0.001) (Supplementary Table S1; Fig. 2B). Specifically, the duration in the medium prey availability and high prey availability groups was significantly lower than in the other treatments, but there was no significant difference between them (Supplementary Table S2; Fig. 2B).

### Web architecture

**Figure 3:**
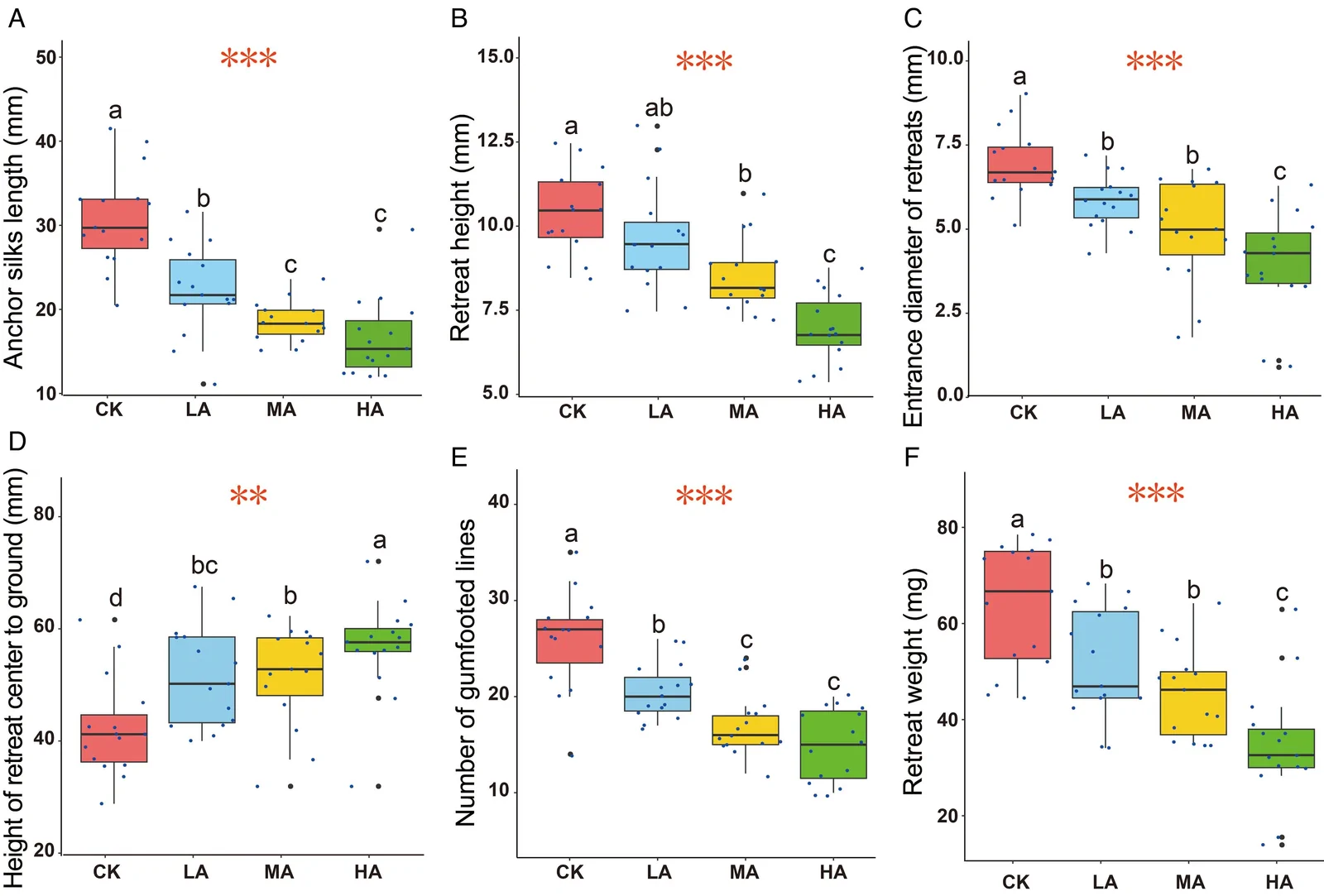
Boxplots of the parameters of web architecture for *C. campanulata* in different availability of ants: (A) Anchor silks length (ASL); (B) retreat height (RH); (C) entrance diameter of retreat (ED); (D) height of retreat center to ground (CRH); (E) number of gumfooted lines (GLN); and (F) retreat weight (RW). Boxplots show the median (central line), first and third quartiles (box), and different lower-case letters indicate a significant difference between treatments. **P < 0.01; ***P < 0.001.

**Figure 4:**
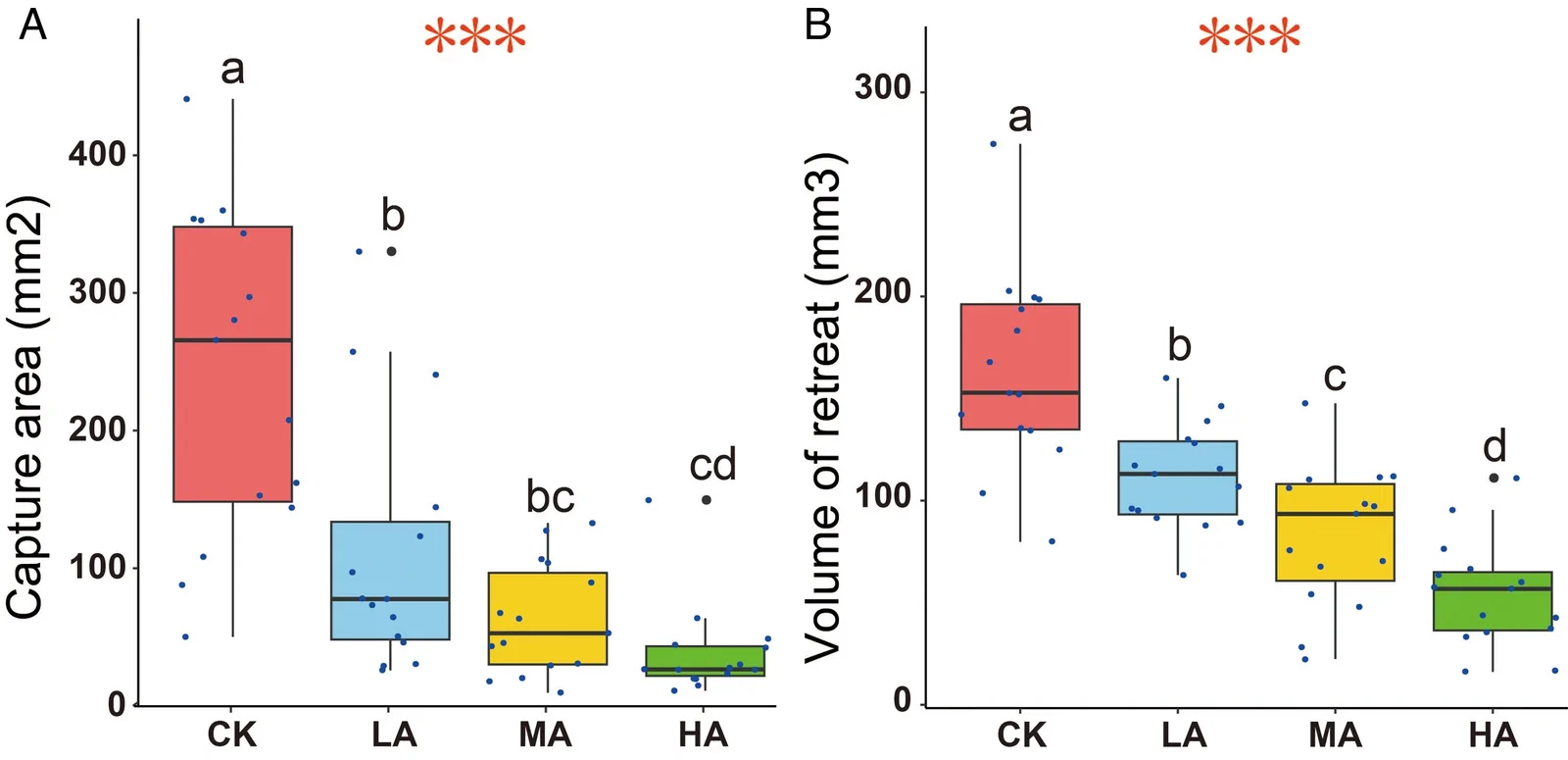
Boxplots showing the capture area and retreat volume for *C. campanulata* under different prey availability conditions (number of ants): (A) Capture area (CA); (B) volume of retreat (RV). Boxplots show the median (central line), first and third quartiles (box), and different lower-case letters indicate significant differences between treatments. ***P < 0.001.

Significant differences were observed in all measured parameters of web architecture among the four treatments (ASL: *χ^2^* = 33.172, *df* = 3, and *P* = 0.019, Supplementary Table S1, Fig. 3A; RH: *χ^2^* = 33.031, *df* = 3, and *P* < 0.001, Supplementary Table S1, Fig. 3B; ED: *χ^2^* = 29.052, *df* = 3, and *P* < 0.001, Supplementary Table S1, Fig. 3C; GLN: *χ^2^* = 32.026, *df* = 3, and *P* < 0.001, Supplementary Table S1, Fig. 3E; RW: *χ^2^* = 27.365, *df* = 3, and *P* < 0.001, Supplementary Table S1, Fig. 3F). Additionally, the capture area (*χ^2^* = 30.868, *df* = 3, and *P* < 0.001, Supplementary Table S1, Fig. 4A) and retreat volume (*χ^2^* = 35.328, *df* = 3, and *P* < 0.001, Supplementary Table S1, Fig. 4B) of *C. campanulata* exhibited significant variation across treatments.

### Behavioral investment

**Figure 5:**
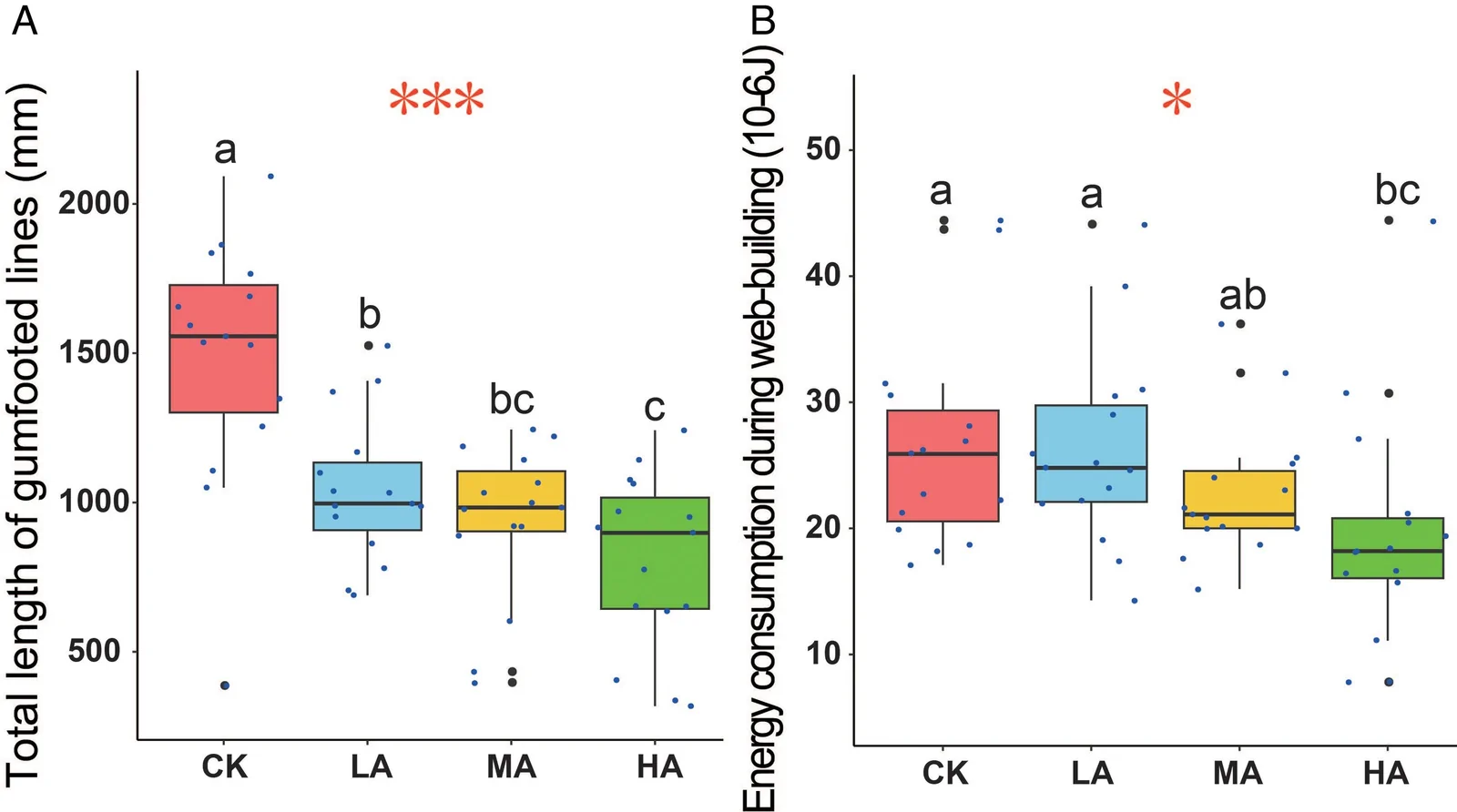
Boxplots showing the foraging investment and defense investment for *C. campanulata* under different prey availability conditions (number of ants): (A) Total length of gumfooted lines (GTL, i.e. foraging investment); (B) energy consumption during retreat-building (EC, i.e. defense investment). Boxplots show the median (central line), first and third quartiles (box), and different lower-case letters indicate a significant difference between treatments. *P < 0.05; ***P < 0.001.

Behavioral investment showed significant differences among treatments (Supplementary Table S1; Fig. 5). Specifically, foraging investment (GTL: *χ^2^* = 23.373, *df* = 3, and *P* < 0.001, Supplementary Table S1, Fig. 5A) and defense investment (EC: *χ^2^* = 35.328, *df* = 3, and *P* = 0.023, Supplementary Table S1, Fig. 5B) exhibited significant variation across the four treatments. These results indicate that high ant availability significantly influenced both foraging and defense investments, with spiders dynamically adjusting their behavioral investments in response to changes in ant abundance.

## Discussion

Our study demonstrated that cobweb spiders can respond to variation in prey availability by adjusting their web architecture through phenotypic plasticity and redistributing investment. The shorter web-building duration indicates that spiders can more quickly escape from dangerous ants and avoid being attacked. Therefore, these results confirm that spiders accelerate web construction in environments with high ant availability. When more ant-prey were present, *Campanicola campanulata* built higher retreats and constructed fewer gumfooted lines. Specifically, webs built by *C. campanulata* under high prey availability had smaller retreats and smaller capture areas compared with those built under low prey availability. Variation in cobweb architecture appears to reflect a reduction in behavioral investment during web-building, likely due to the energy costs associated with constructing gumfooted lines and retreats (Blamires et al. 2014). Meanwhile, lowering the duration of web-building and reducing web-building activity (e.g. constructing smaller retreats and fewer gumfooted lines) may be a strategy to avoid dangerous prey such as ants, which possess strong spines, mandibles, and formic acid for effective group attacks (Pekár and Toft 2015). Except for the latency of web-building, most of the measured parameters in this study exhibited statistically significant differences between treatments. One possible explanation is that housing *C. campanulata* in a smaller web-building device more effectively stimulated changes in the spiders’ web architecture. The very detection of these significant differences provides strong evidence for the adequacy of the experimental setup. Given that web-building spiders are known for their ability to adjust web structure and often build similar webs in both extreme and normal environments (Vollrath et al. 1997), the fact that our treatments yielded distinct results demonstrates that the web-building device was sufficiently large to capture the full plasticity of *C. campanulata*'s web-building behavior.

According to the tradeoff theory, trap-building predators should make decisions based on their physical condition and the costs and benefits of adopting specific behaviors to maximize fitness (Scharf et al. 2011). In our study, we did not find a clear tradeoff between foraging and defense as prey availability increased. The behavioral investment of spiders with high ant availability during web-building was consistent with theoretical predictions: the total length of gumfooted lines (i.e. foraging investment) and energy consumption (i.e. defense investment) were significantly lower than those in other treatments. The trial spiders in our study did not significantly strengthen the construction of foraging structures with increasing prey availability, as observed in other species studies (e.g. fish, Holbrook and Schmitt 1988; antlion, Farji-Brenera and Amador-Vargas 2019). Previous studies used non-lethal flying insects as prey, whereas ants serve as both prey and potential predators for spiders (Pekár and Toft 2015). Additionally, cobweb spiders typically exhibit significant structural variations in defensive retreats and foraging gumfooted lines, which are often mediated by the spider's starvation state (Blamires et al. 2014). However, the trial spiders in our study were well-fed before the experiment and thus lacked the internal drive to express extended phenotypic plasticity fully.

Zhang and colleagues have found that *C. campanulata* spends only about one-tenth of its web-building duration on transporting detritus and building retreats, with the spider resting for most of the time (Zhang et al. 2023c). These results suggest that retreat construction in *C. campanulata* is energetically costly, as they need to expend considerable energy to pull sand grains into the air and use a significant amount of sticky silk to bind them together (Henschel and Jocqué 1994). Zhang et al. also observed that marginally well-fed spiders build smaller retreats in previous studies (Zhang et al. 2023b), even though this increases the risk of the spider being detected and attacked by predators (Manicom et al. 2008). This behavior may be partially explained by the fact that web-building spiders can deposit a pyrrolidine alkaloid (2-pyrrolidone) on their silk, which provides protection from ant invasion (Zhang et al. 2011). Additionally, the configuration of the suspended retreat and the presence of silken tunnels inside significantly reduce the spider's vulnerability to enemies and prevent them from being invaded (Henschel and Jocqué 1994). Therefore, it is not surprising that our well-fed trial spiders did not build large retreats. Furthermore, our results showed that both the total length of gumfooted lines and the energy consumption during retreat construction in *C. campanulata* were significantly reduced in the presence of high ant availability, with no obvious tradeoff. These adjustments to web-building behavior suggest a state-dependent change in web-building strategy.

Previous research has shown that web-building spiders modify their web architecture in response to variation in the type or availability of insect prey (Nakata 2009; Blamires 2010; DiRienzo and Montiglio 2016; Sergi et al. 2020; Thompson et al. 2020). These modifications are often associated with changes in the spiders’ body condition (Blackledge and Zevenbergen 2007; Zevenbergen et al. 2008; Zhang et al. 2023b) and internal nutritional status (Baba and Miyashita 2006; Blamires 2013). Such variations in functional components are expected to significantly influence investment decisions during cobweb construction, thereby affecting behavioral investment strategies, as different components impact the foraging and defense functions of the cobweb. Consistent with the findings of DiRienzo and Montiglio (2016) and Toupin et al. (2022), our results indicate that cobweb spiders exhibit flexibility in adjusting their web architectures in response to the prey environment. This flexibility is evident in their ability to make pronounced adjustments, such as constructing larger retreats or increasing the number of gumfooted lines, as observed in this study.

Sergi et al. (2020) reported that there is no plasticity in the response of cobweb spiders to variation in the prey environment, noting that black widows do not adjust their web architecture according to environmental conditions. However, our study on *C. campanulata* revealed that when encountering more dangerous ant-prey, the spiders built retreats that were one-third smaller than those built in the absence of ants. Additionally, spiders in the high ant availability treatment exhibited a slight trend toward constructing webs with smaller capture areas and fewer gumfooted lines, which was not associated with increased foraging success. One possible explanation here could be that reduced investment in web construction can also be due to the possibility of vacating the web location under extreme circumstances. Furthermore, we found that the effect of prey availability was still evident when analyzed separately for foraging investment and defense investment.

Numerous studies have confirmed that plastic adjustments in web architecture and behavioral investment are triggered by the spider's starvation state, allowing spiders to actively manipulate the volume or weight of retreats and the number or total length of gumfooted lines under different conditions (Blackledge and Zevenbergen 2007; Zevenbergen et al. 2008; Toupin et al. 2022; Zhang et al. 2023b). This is because the parameters of retreat are proportional to defense investment, whereas the number and total length of gumfooted lines are proportional to foraging investment (Zhang et al. 2023b; Fischer et al. 2024). Therefore, the observed variations in retreat and gumfooted lines in cobwebs can be interpreted as spiders actively adjusting their web architecture and behavioral investment in response to prey availability.

Our research directly confirmed the previous findings that cobweb spiders utilize various cues from their prey and their own condition to adjust different functional components of the web and reallocate behavioral investment during web-building to optimally allocate limited resources. Sherman (1994) reported that in *Larinioides cornutus*, satiated spiders redistributed energy from continued foraging into laying eggs, whereas hungry spiders invested more energy in foraging. Zhang et al. (2023a) found that mature female *C. campanulata* devoted more energy to defense and less to foraging. Zhang et al. (2023b) discovered that marginally well-fed *C. campanulata* built cobwebs with a greater number and longer gumfooted lines, as well as a larger capture area to enhance foraging, whereas extremely well-fed spiders built cobwebs with a significantly larger retreat volume to strengthen their defenses. These findings are consistent with the results of our study. However, we did not test the effects of egg production and hatching in post-mating females on cobweb architecture. Previous research has shown that adult female cobweb spiders alter their cobweb architecture at the expense of safety and foraging when they have egg sacs (DiRienzo and Aonuma 2018; Zhang et al. 2023a). These potential factors could be investigated in future studies.

In summary, we tested the extended phenotypic plasticity of *C. campanulata* under different levels of prey availability. The results showed that ant availability significantly affected the cobweb architecture of *C. campanulata*. Under high prey availability, spiders reduced their investment in both foraging and defense, but there was no significant tradeoff between these two forms of investment. This behavior may represent an adaptive strategy that has evolved to optimize resource allocation based on the spider's internal state and to prioritize the reduction of the risk of being detected by predators.

## Supplementary Material

zoaf073_Supplementary_Data

## Data Availability

All the data supporting the results can be archived at https://doi.org/10.5061/dryad. dz08kps7p.
