# Supplementary material for: Phenotypic plasticity in an extended phenotype: the cobweb spider Campanicola campanulata alters web architecture in response to variation in prey availability
Source: Curr Zool. 2025 Nov 19;72(4):590–9. doi: 10.1093/cz/zoaf073 (PMC13500169; doi:10.1093/cz/zoaf073)
Supplement: Supplementary file 1 [file zoaf073_supplementary_data.zip › Table S1.docx]

**Table S1.** Mean(±SE) of all parameters measured in study and results of Kruskal-Wallis rank sum test between treatments (N=15 in each treatment).

| **Parameters** | **Treatment** | | | | ***χ^2^*** | ***df*** | ***P*** |
| --- | --- | --- | --- | --- | --- | --- | --- |
|  | **CK** | **LA** | **MA** | **HA** |  |  |  |
| **LW** | 8.922±1.780 | 9.017±1.902 | 9.067±1.990 | 10.033±1.875 | 3.258 | 3 | 0.354 |
| **DW** | 21.047±5.965 | 16.499±2.337 | 15.481±2.004 | 15.415±2.373 | 28.01 | 3 | < 0.001 |
| **ASL** | 30.900±5.874 | 22.280±5.354 | 18.547±2.359 | 16.613±4.730 | 33.172 | 3 | < 0.001 |
| **RH** | 10.313±1.256 | 9.567±1.590 | 8.473±1.093 | 6.907±1.017 | 33.031 | 3 | < 0.001 |
| **ED** | 7.353±1.027 | 6.253±0.776 | 5.360±1.514 | 4.407±1.537 | 29.052 | 3 | < 0.001 |
| **CRH** | 42.293±8.841 | 51.633±8.989 | 51.653±8.929 | 56.580±8.799 | 14.689 | 3 | 0.002 |
| **GLN** | 25.800±5.060 | 20.533±2.900 | 16.933±3.195 | 14.867±3.796 | 32.026 | 3 | < 0.001 |
| **RW** | 63.807±12.872 | 51.607±11.474 | 45.507±9.464 | 34.653±12.339 | 27.365 | 3 | < 0.001 |
| **RV** | 149.689±47.906 | 98.560±25.731 | 69.460±34.675 | 40.827±26.543 | 35.328 | 3 | < 0.001 |
| **CA** | 240.340±118.677 | 111.129±93.207 | 62.680±40.519 | 38.260±33.752 | 30.868 | 3 | < 0.001 |
| **GTL** | 1484.047±415.365 | 1040.389±246.133 | 934.233±264.011 | 802.413±293.586 | 23.373 | 3 | < 0.001 |
| **EC** | 26.487±8.383 | 26.167±7.822 | 22.760±5.457 | 19.553±9.214 | 9.557 | 3 | 0.023 |

LW: the latency of web-building; DW: the duration of web-building; ASL: the length of anchor silk; RH: the height of retreat; ED: the diameter of retreat entrance; CRH: the height of retreat center from the ground; GLN: the number of gumfooted line; RW: the weight of retreat; RV: the volume of retreat; CA: capture area of the web; GTL: the total length of gumfooted line; EC: the energy consumption during web-building.
