# Supplementary material for: Phenotypic plasticity in an extended phenotype: the cobweb spider Campanicola campanulata alters web architecture in response to variation in prey availability
Source: Curr Zool. 2025 Nov 19;72(4):590–9. doi: 10.1093/cz/zoaf073 (PMC13500169; doi:10.1093/cz/zoaf073)
Supplement: Supplementary file 1 [file zoaf073_supplementary_data.zip › Table S2.docx]

**Table S2.** Results of a paired comparison of the Wilcoxon rank sum test with Bonferroni correction for parameters with significant differences.

| **Parameters** | **CK VS LA** | | **CK VS MA** | | **CK VS HA** | | **LA VS MA** | | **LA VS HA** | | **MA VS HA** | |
| --- | --- | --- | --- | --- | --- | --- | --- | --- | --- | --- | --- | --- |
|  | ***W*** | ***P*** | ***W*** | ***P*** | ***W*** | ***P*** | ***W*** | ***P*** | ***W*** | ***P*** | ***W*** | ***P*** |
| **DW** | 174 | 0.068 | 214.5 | < 0.001 | 186 | 0.015 | 195.5 | 0.004 | 101 | 1.000 | 193.5 | 0.005 |
| **ASL** | 195.5 | 0.004 | 222 | < 0.001 | 216 | < 0.001 | 172 | 0.087 | 46 | 0.037 | 64 | 0.278 |
| **RH** | 153 | 0.579 | 191.5 | 0.007 | 223 | < 0.001 | 158.5 | 0.354 | 12.5 | <0.001 | 31.5 | 0.005 |
| **ED** | 183 | 0.022 | 197.5 | 0.003 | 218.5 | < 0.001 | 150.5 | 0.717 | 29.5 | 0.004 | 64.5 | 0.292 |
| **CRH** | 46 | 0.034 | 52 | 0.077 | 34 | 0.007 | 106.5 | 1.000 | 151.5 | 0.661 | 148.5 | 0.844 |
| **GLN** | 189 | 0.009 | 205.5 | 0.001 | 216 | < 0.001 | 186.5 | 0.013 | 30 | 0.004 | 86 | 1.000 |
| **RW** | 173.5 | 0.072 | 192.5 | 0.006 | 215 | < 0.001 | 141 | 1.000 | 30 | 0.004 | 50 | 0.061 |
| **RV** | 187 | 0.008 | 208 | < 0.001 | 222 | < 0.001 | 165 | 0.177 | 14.5 | < 0.001 | 57 | 0.125 |
| **CA** | 187 | 0.008 | 210 | < 0.001 | 220 | < 0.001 | 147 | 0.964 | 33 | 0.004 | 67 | 0.368 |
| **GTL** | 195 | 0.002 | 201 | 0.001 | 206 | < 0.001 | 130 | 1.000 | 62 | 0.220 | 79 | 1.000 |
| **EC** | 114 | 1.000 | 142.5 | 1.000 | 172.5 | 0.081 | 148 | 0.879 | 55 | 0.108 | 67 | 0.371 |

DW: the duration of web-building; ASL: the length of anchor silk; RH: the height of retreat; ED: the diameter of retreat entrance; CRH: the height of retreat center from the ground; GLN: the number of gumfooted lines; RW: the weight of retreat; RV: the volume of retreat; CA: capture area of the web; GTL: the total length of gumfooted lines; EC: the energy consumption during retreat-building.
